# A Machine Learning-Based Screening Test for Sarcopenic Dysphagia Using Image Recognition

**DOI:** 10.3390/nu13114009

**Published:** 2021-11-10

**Authors:** Kotomi Sakai, Stuart Gilmour, Eri Hoshino, Enri Nakayama, Ryo Momosaki, Nobuo Sakata, Daisuke Yoneoka

**Affiliations:** 1 Graduate School of Public Health, St. Luke’s International University, Tokyo 104-0044, Japan; ko-sakai@fc.ritsumei.ac.jp (K.S.); sgilmour@slcn.ac.jp (S.G.); 2 Setagaya Memorial Hospital, Tokyo 158-0092, Japan; sakata.nobuo@hmw.gr.jp; 3 Comprehensive Unit for Health Economic Evidence Review and Decision Support (CHEERS), Research Organization of Science and Technology, Ritsumeikan University, Kyoto 600-8815, Japan; hoshieri@fc.ritsumei.ac.jp; 4 Department of Dysphagia Rehabilitation, Nihon University School of Dentistry, Tokyo 101-8310, Japan; nakayama.enri@nihon-u.ac.jp; 5 Department of Rehabilitation Medicine, Mie University Graduate School of Medicine, Tsu 514-8407, Japan; momosakiryo@gmail.com; 6 Department of Health Services Research, Faculty of Medicine, University of Tsukuba, Tsukuba 305-8575, Japan; 7 Department of Health Policy and Management, School of Medicine, Keio University, Tokyo 106-8582, Japan; 8 Department of Global Health Policy, Graduate School of Medicine, The University of Tokyo, Tokyo 113-0033, Japan; 9 Tokyo Foundation for Policy Research, Tokyo 106-6234, Japan

**Keywords:** dysphagia, sarcopenia, screening, image recognition

## Abstract

Background: Sarcopenic dysphagia, a swallowing disorder caused by sarcopenia, is prevalent in older patients and can cause malnutrition and aspiration pneumonia. This study aimed to develop a simple screening test using image recognition with a low risk of droplet transmission for sarcopenic dysphagia. Methods: Older patients admitted to a post-acute care hospital were enrolled in this cross-sectional study. As a main variable for the development of a screening test, we photographed the anterior neck to analyze the image features of sarcopenic dysphagia. The studied image features included the pixel values and the number of feature points. We constructed screening models using the image features, age, sex, and body mass index. The prediction performance of each model was investigated. Results: A total of 308 patients participated, including 175 (56.82%) patients without dysphagia and 133 (43.18%) with sarcopenic dysphagia. The area under the receiver operating characteristic curve (ROC-AUC), sensitivity, specificity, positive predictive value, negative predictive value, and area under the precision-recall curve (PR-AUC) values of the best model were 0.877, 87.50%, 76.67%, 66.67%, 92.00%, and 0.838, respectively. The model with image features alone showed an ROC-AUC of 0.814 and PR-AUC of 0.726. Conclusions: The screening test for sarcopenic dysphagia using image recognition of neck appearance had high prediction performance.

## 1. Introduction

The global population is aging. The proportion of individuals aged 65 years and over was 9.3% in 2020, and this figure is expected to almost double by 2050 [1]. Given the aging population, sarcopenia, a geriatric syndrome characterized by generalized loss of muscle mass and function mainly caused by aging, malnutrition, and inactivity, has gained attention as a possible leading cause of the loss of physical independence and all-cause death [2,3]. Sarcopenia can also cause a decline in swallowing function [4]. Dysphagia (swallowing difficulties) due to sarcopenia is defined as “sarcopenic dysphagia” and is a new concept in the field of geriatric medicine [5,6]. The prevalence of sarcopenic dysphagia among hospitalized older adults has been reported to be 32% in the acute setting and 35% in the post-acute setting [7,8]. Dysphagia is known to be a leading cause of malnutrition, aspiration pneumonia, choking, and death [9]. Therefore, early detection and effective interventions are essential to prevent these consequences.

There are some possible indices for the diagnosis of sarcopenic dysphagia [10]. However, screening tests for sarcopenic dysphagia have not been investigated. Most existing screening tests for dysphagia have been developed primarily for stroke patients [11]. However, in clinical practice, they have been applied to other populations [12]. The mismatch between test development and the target population can make screening ineffective and inefficient. Currently, most screening tests include a water swallow test, the assessment of oral movements, or voluntary cough [13]. These test components present a risk of droplet spread of respiratory infections such as coronavirus disease 2019 (COVID-19). Due to the fears associated with the COVID-19 pandemic, clinicians may be uncomfortable administering those screening tests and avoid them. In addition, choking during the water swallow test can be painful for patients with sarcopenic dysphagia due to their low respiratory strength [14]. Questionnaire-based screening tests are potential diagnostic alternatives. However, older individuals may not be aware of their swallowing dysfunction [15]. In addition, the reliability of questionnaires may be compromised, especially in older individuals with cognitive impairment [16]. Furthermore, tests should be administered by trained health professionals to ensure accurate assessment.

Based on these issues, a screening test that can be performed easily with a low risk of droplet spread is needed for the early identification of cases of sarcopenic dysphagia. Muscles in the neck are strongly involved in swallowing function. A study reported that a smaller neck circumference, which implies decreased muscle mass, is associated with weaker swallowing-related muscle strength [17]. Since decreased muscle mass is a major component of sarcopenia, patients with sarcopenic dysphagia may have a characteristic neck appearance due to loss of neck muscle mass. The objectives of this study were to develop a simple and noninvasive screening test for sarcopenic dysphagia with low risk of droplet transmission using machine learning techniques (in particular the image recognition of neck appearance), and to investigate the prediction performance of the tests in older patients.

## 2. Materials and Methods

### 2.1. Study Population

Consecutive older patients admitted to a hospital for post-acute rehabilitation in Tokyo, Japan, between February 2020 and November 2020, participated in this cross-sectional study. The inclusion criteria were as follows: (1) age ≥ 65 years; and (2) Glasgow Coma Scale score of 14 or 15 (4 in eye-opening, 4 or 5 in best verbal response, and 6 in best motor response). The exclusion criteria were as follows: (E1) history of dysphagia following stroke or brain injury, or onset of stroke or brain injury within the past 6 months; (E2) underlying degenerative neurological diseases; (E3) history of head and neck cancer; (E4) connective tissue diseases; (E5) unstable general condition; (E6) past or current tracheostomy; (E7) having a beard on the anterior triangle of the neck; and (E8) carrier of pacemakers or implantable cardiac defibrillators. Criteria E1–5 excluded patients with definitive causes of dysphagia not due to sarcopenia [10,18]. In other words, our screening test targets people who do not have a disease that can cause dysphagia other than sarcopenia.

### 2.2. Diagnosis of Sarcopenic Dysphagia

Sarcopenic dysphagia was diagnosed using the diagnostic criteria described in a position paper by independent medical doctors within 1 week of hospitalization [10]. The criteria are used to determine 3 diagnostic categories: (1) definite diagnosis, defined by dysphagia, generalized sarcopenia, loss of swallowing muscle mass, and no other causes except sarcopenia; (2) probable diagnosis, defined by dysphagia, generalized sarcopenia, and no other causes for dysphagia except sarcopenia; and (3) possible diagnosis, defined by dysphagia, generalized sarcopenia, and no definitive cause of dysphagia. We used criterion 3 (a possible diagnosis) for our study. In addition, we used low lingual pressure as a diagnosis criterion because it is a possible characteristic of sarcopenic dysphagia [10]. Sarcopenia was diagnosed based on the Asian Working Group for Sarcopenia 2019 algorithm [19]. In this algorithm, individuals with low muscle mass, reduced muscle strength, or reduced physical function are diagnosed as having sarcopenia. As our study included patients with orthopedic disorders, we did not use the physical function as a diagnostic criterion. Low handgrip strength was assessed using a digital grip strength dynamometer (TKK 5401; Takei Scientific Instruments, Tokyo, Japan) to provide an index for the reduction of muscle strength (men: <28 kg, women: <18 kg). Participants were asked to sit in an upright position and grip the instrument 3 times with each hand. The highest value was used for the diagnosis. Low skeletal muscle mass index was assessed using bioimpedance analysis (men: <7.0 kg/m^2^, women: <5.7 kg/m^2^) (InBody S10, InBody Japan Inc., Tokyo, Japan). Dysphagia was diagnosed when all the following criteria were met: (1) positive results in the 100 mL or modified water swallow test as screening tests for dysphagia [20,21]; (2) need for texture-modified food or liquid as assessed by speech-language therapists, or no food intake by mouth; and (3) abnormal findings regarding swallowing safety in the videofluoroscopic swallowing study (VFSS), which is a gold standard tool to assess dysphagia. We used volumes of 3, 5, or 10 mL, and/or sips from a cup of thickened and/or thin barium sulfate solution mixed with water (40% weight/volume) in the VFSS. We scored each swallow using the penetration–aspiration scale, which ranged from 1 (material did not enter the airway) to 8 (material entered the airway, passed below the level of the vocal folds, and no effort was made to eject) and considered patients with scores of 3 (material entered the airway, remained above the vocal folds, and was not ejected from the airway) and over as abnormal [22]. Regarding the measurement of the lingual pressure, no consensus exits for defining the cut-off value for possible sarcopenic dysphagia [10]. Therefore, in our study, the low lingual pressure was set at a cut-off value of 30 kPa, as defined in the standardized criterion used in Japan [23], and was measured by speech-language therapists. We measured lingual pressure 3 times using a balloon-type disposable probe (JMS, Hiroshima, Japan), which measures the pressure of the anterior tongue. The highest value among the 3 trials was used as the lingual pressure. We defined patients without sarcopenic dysphagia as being non-dysphagia patients. Because not all people with sarcopenia develop dysphagia, we included patients even with sarcopenia in the non-dysphagic group.

### 2.3. Neck Imaging

**Figure 1:**
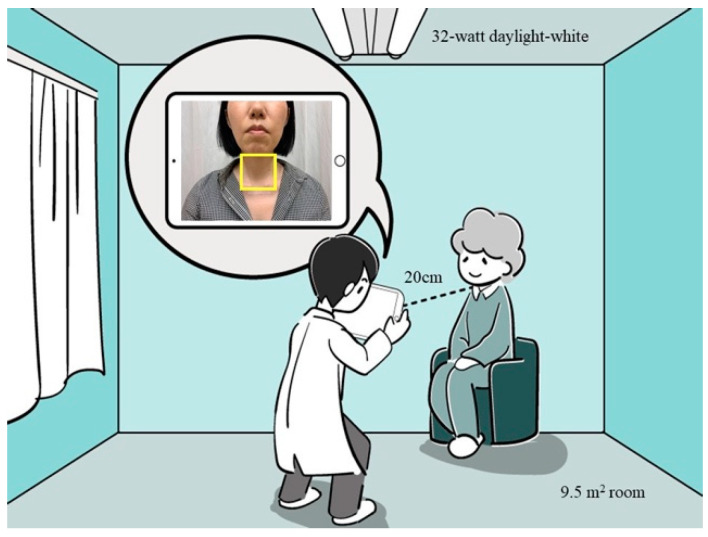
Environment used for taking a photo and the cropping area of the image. The cropping area is indicated by yellow square.

We focused on the anterior lower neck. A protruding clavicle is a sign of loss of muscle mass, which also presents as recessed areas around the clavicle [24]. Therefore, we hypothesized that images of the anterior lower neck would be informative for detecting sarcopenic dysphagia. A photograph of the neck was taken in a standardized environment on the day of admission by trained research staff (Figure 1). Patients were asked to sit upright in a chair or in a wheelchair with their chin in a neutral position as much as possible. Surgical tape was placed at the upper end of the breastbone. A photo of the neck was taken using an iPad (model A1474, Apple, Inc., Los Altos, CA, USA) at a distance of 20 cm from the tip of the chin. The top line of the image was aligned under the eyes horizontally. The room in which the photo was taken was 9.5 m^2^ in size, illuminated by two 32-watt daylight-white fluorescent tubes without direct sunlight.

The image was preprocessed as follows: (1) the image was cropped considering the lower end of the jawline as the upper edge, the maximum horizontal length of the neck without the background, and the tape on the breastbone as the lower edge; (2) the bottom half of the cropped image was extracted; (3) the image was converted to grayscale; and (4) a median filter with a 5 × 5 pixel kernel size was applied to remove speckling noise [25]. Step 1 was processed manually on the iPad using the crop function directly, and steps 2–4 were performed using the EBImage package in R (version 4.0.3).

### 2.4. Image Features

We used the median and interquartile range (IQR) of the pixel values and the number of feature points per pixel as the image features. The pixel value represents the brightness of the pixel, and higher pixel values indicate higher brightness (i.e., 1 is white and 0 is black). Feature points of an image were estimated using the Features from Accelerated Segment Test (FAST) algorithm in the OpenCV package in R [26]. The FAST algorithm detects corners (i.e., edges) as feature points in an image at a high speed automatically. It takes only a few seconds to process an image using a personal computer (MacBook pro, Processor 2.3 GHz quad-core Intel Core i7, Memory 16 GB). We used the algorithm to capture the boundaries of the recessed area and wrinkles caused by the loss of muscle mass on the neck, which were detected as corners. A higher number of feature points indicated more corners. The neighborhood radius was set to 3 and the brightness threshold was set to 2.5 in the FAST algorithm by our visual judgement.

### 2.5. Participant Characteristics

We used the Charlson Comorbidity Index for comorbid conditions [27], the Barthel Index for activities of daily living [28], the Functional Oral Intake Scale (FOIS) for texture levels of food and liquid [29], and the Mini-Mental State Examination for cognitive status [30]. The FOIS score ranged from levels 7 to 1. Levels 7 and 6 did not require special food or liquid modification. However, levels 5 and 4 required special food or liquid modification to be taken orally. Levels 3 and under were tube-dependent. Malnutrition was assessed using the Global Leadership Initiative on Malnutrition criteria [31].

### 2.6. Statistical Analysis

Subjects were randomly assigned to training data (70%) and test data (30%) groups [32]. To construct the screening models, body mass index (BMI) was categorized into 2 groups: underweight (BMI < 18.5 kg/m^2^) and not underweight (BMI ≥ 18.5) [33], to allow for practical visual assessment of the body shape, assuming that the exact BMI at the point of screening would be unknown or may not be immediately available in many situations, and to simplify screening [34].

Three screening models were constructed using the training data. Model 1 used sex, BMI categories, and all image features. Model 2 used age, sex, BMI categories, and all image features. Model 3 used age, sex, BMI categories, and all image features except for the median pixel value. We assumed a practical situation in Model 1 with no access to information on age; thus, Model 1 used a minimal set of covariates obtained only from the patient’s appearance. In Model 2, the assumption was relaxed to assume the availability of age information. In Model 3, we removed the covariate of the median pixel value from Model 2, a value that could be most affected by the brightness of the room, to avoid an assessment that might highly depend on the environment. We also tested models with the same set of covariates, except for the image features, and a model with only the image features to investigate the impact of image features on prediction performance.

We used the independent *t*-test for continuous variables and the chi-squared test or Fisher’s exact test for categorical variables to compare characteristics between the non-dysphagic group and the sarcopenic dysphagic group. Kernel density estimation with a Gaussian kernel was used to illustrate the probabilistic density curves of the image features. A logistic regression model was used to model the binary outcomes of non-dysphagia or sarcopenic dysphagia. All continuous covariates in the models were standardized. To select an optimal set of features, we used least absolute shrinkage and selection operator (LASSO) regression with 10-fold cross-validation to find an optimal smoothing parameter for each model [35]. The area under the receiver operating characteristics curve (ROC-AUC), sensitivity, specificity, positive predictive value (PPV), negative predictive value (NPV), and area under the precision-recall curve (PR-AUC) were used to evaluate the prediction performance of the test data. To calculate sensitivity, specificity, PPV, and NPV, a threshold value for binarizing the estimated probability was calculated based on the Youden index approach [36]. A 2-sided *p* value of <0.05 was considered statistically significant. *R* (version 4.0.3) was used for the analysis.

## 3. Results

A total of 504 patients were recruited in this study. After applying the exclusion criteria, 308 patients participated (male: 128 (41.56%)) in the final analysis, with 175 (56.82%) patients in the non-dysphagic group and 133 (43.18%) patients in the sarcopenic dysphagic group. The mean age (standard deviation (SD)) was 84.15 (8.01) years, and the most prevalent primary disease for admission was an orthopedic disorder (57.47%). Femoral neck fractures accounted for 70.62% of these orthopedic disorders. The prevalence of sarcopenia was 76.95%, with 59.43% in the non-dysphagic group and 100% in the sarcopenic dysphagic group. The mean FOIS (SD) was 6.60 (0.49) and 3.84 (1.60) in the non-dysphagic and sarcopenic dysphagic groups, respectively. Regarding the FOIS in the sarcopenic dysphagic group, level 5 was 57.14%, level 4 was 15.04%, and under level 4 was 27.82%. Patient characteristics are shown in Table 1.

**Figure 2:**
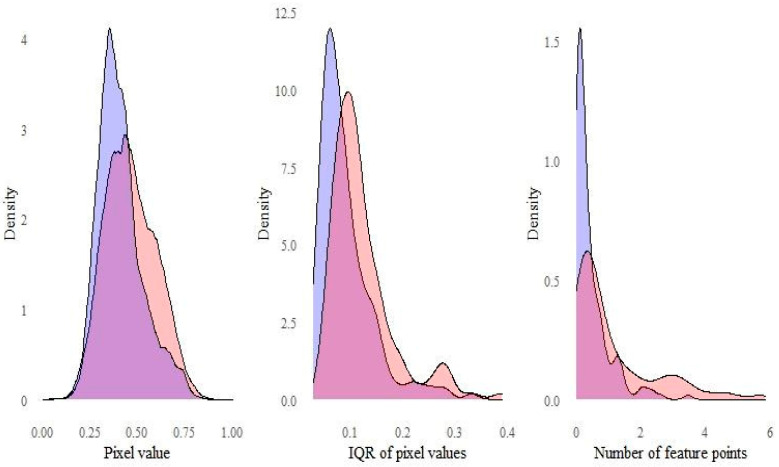
Kernel density curve for image features. Blue = non-dysphagic group, Red = sarcopenic dysphagic group.

**Figure 3:**
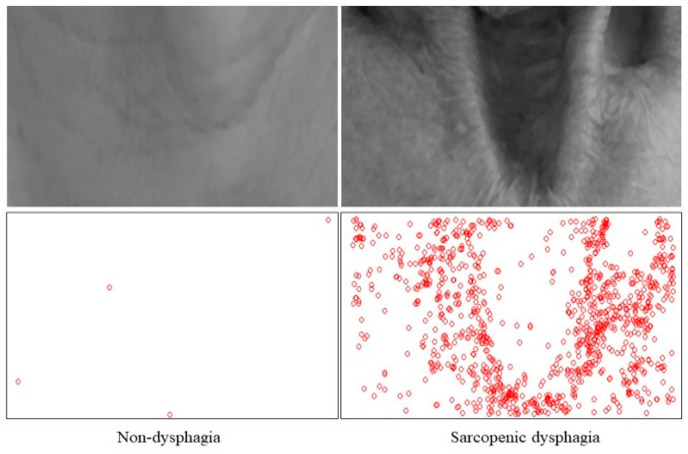
Gray scale images and relative feature points detected by the Features from Accelerated Segment Test (FAST) algorithm.

The image feature values in the sarcopenic dysphagic group were significantly greater than those in the non-dysphagic group (*p* < 0.001 for all). The mean median pixel (SD) values were 0.40 (0.09) and 0.45 (0.11), the mean IQR of pixel values were 0.09 (0.05) and 0.12 (0.06), and the mean numbers of feature points per pixel were 0.44 (0.56) and 1.10 (1.28) in the non-dysphagic and the sarcopenic dysphagic groups, respectively. The kernel density curves for pixel values, the IQR of pixel values, and the number of feature points are shown in Figure 2. Typical grayscale images, with the median filter of a non-dysphagic patient and sarcopenic dysphagic patient and their feature points detected by the FAST algorithm, are shown in Figure 3.

**Figure 4:**
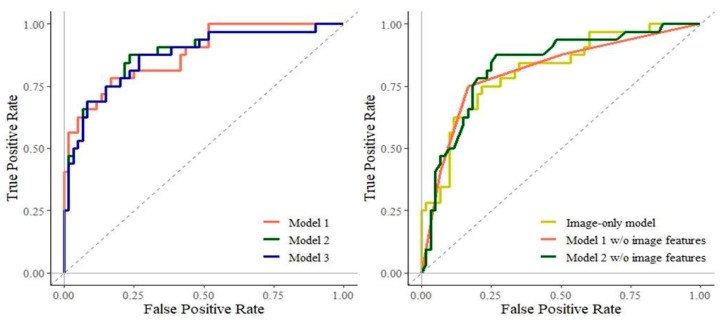
Receiver operating characteristic curves characterizing the screening models. Model 1: sex, categorized BMI, median pixel value, IQR of pixel value, number of feature points. Model 2: age, sex, categorized BMI, median pixel value, IQR of pixel value, number of feature points. Model 3: age, sex, categorized BMI, IQR of pixel value, number of feature points. Image-only model: median pixel value, IQR of pixel value, number of feature points. Abbreviations: BMI, body mass index; IQR, interquartile range; w/o = without.

**Figure 5:**
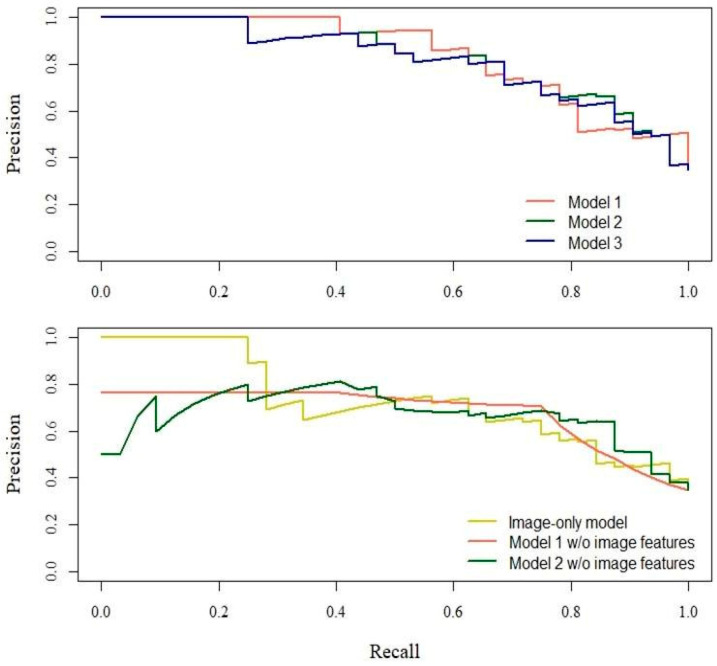
Precision-recall curves for the screening models. Model 1: sex, categorized BMI, median pixel value, IQR of pixel value, number of feature points. Model 2: age, sex, categorized BMI, median pixel value, IQR of pixel value, number of feature points. Model 3: age, sex, categorized BMI, IQR of pixel value, number of feature points. Image-only model: median pixel value, IQR of pixel value, number of feature points. Abbreviations: BMI, body mass index; IQR, interquartile range; w/o = without.

After LASSO variable selection, no covariates were dropped. The estimated odds ratios (ORs) for each variable are shown in Table 2. The OR of the number of feature points was the highest among the image features: 1.65 (95% confidence interval (CI): 1.06–2.56) in Model 1; 1.55 (95% CI: 0.99–2.41) in Model 2; and 1.61(95% CI: 1.07–2.41) in Model 3. The prediction performances of the models are shown in Table 3. The ROC-AUC, sensitivity, specificity, PV, NPV, and PR-AUC values were 0.877, 87.50%, 76.67%, 66.67%, 92.00%, and 0.838, respectively, in the best model (Model 2). Models 1 and 2 without image features showed lower ROC-AUC and PR-AUC than those with image features. In the image feature-only model, ROC-AUC, sensitivity, specificity, PPV, NPV, and PR-AUC values were 0.814, 71.88%, 80.00%, 65.71%, 84.21%, and 0.726, respectively. The ROC curves and PR curves for the models are shown in Figure 4 and Figure 5, respectively.

## 4. Discussion

This study developed a simple and noninvasive machine-learning-based screening test using image recognition of the neck appearance. This test showed high prediction performance for the identification of sarcopenic dysphagia.

Compared with the existing screening tests for dysphagia, which are also used for people with risk of sarcopenic dysphagia, our screening test is the first objective screening test that can be conducted without direct invasion of the mouth or pharynx, thus avoiding the possibility of choking and risk of droplet spread. Screening tests should be acceptable and cause minimal discomfort during their performance [37]. Our test may represent a more acceptable alternative with less discomfort for both patients and the clinicians who administer it. Among the existing water swallow tests, consecutive sips of 90 to 100 mL of water have been reported as having a highest pooled sensitivity of 91% and specificity of 53% in a recent meta-analysis [38]. Our models showed relatively better prediction performance with a lower risk of droplet transmission. Nevertheless, a screening test should be easy and quick [37]. Analyzing image features requires multiple steps and may be time consuming. Therefore, developing an advanced application that can automatically process images at high speeds would be useful.

In the comparison of models with and without image features, the model including the image features presented a higher ROC-AUC and PR-AUC. Consistent with previous results, our study showed that the neck appearance was significantly associated with sarcopenic dysphagia and contained useful predictive information [17]. Among the image features, the OR of the number of feature points was significant in models 1 and 3. Although other image features were not associated with a significant OR in the models, all image features remained as potential predictors after LASSO-based variable selection. In addition, the model based on only image features had a good prediction performance. These results implied that image features have a significant impact on prediction [39]. A larger number of feature points in patients with sarcopenic dysphagia indicates that the boundaries of the recessed area and wrinkles caused by decreased muscle mass on the neck could be detected as corners or edges using the FAST algorithm.

This study presents some limitations. First, this study was conducted at a single center. Therefore, our data might have included a selection bias. Second, although the images of the neck were taken in a standardized environment, we did not check the prediction performance with different environments or light sources. Therefore, our data may have included measurement bias and generalizability might be limited. Finally, we used the FAST algorithm to detect the boundaries of the recessed area and wrinkles caused by the loss of muscle mass in the neck. Its precise validity is unclear, but our results show that the FAST algorithm can be useful for detecting sarcopenia.

## 5. Conclusions

We showed that a machine learning-based screening test using image recognition analysis of the neck appearance is useful for screening for sarcopenic dysphagia, with high prediction performance. As implications, our novel screening test may facilitate the screening of sarcopenic dysphagia using a simple image of the neck even during the COVID-19 pandemic.

## Figures and Tables

**Table 1:** Characteristics of study participants.

	Non-Dysphagic Group (*n* = 175)	Sarcopenic Dysphagic Group (*n* = 133)	*p* Value
Age (years)	82.57 (8.01)	86.22 (7.47)	<0.001
Sex; female, No. (%)	108 (62.43)	72 (54.15)	0.20
Sarcopenia, No. (%)	104 (59.43)	133 (100.00)	<0.001
C-reactive protein (mg/dL)	0.40 (0.60)	0.87 (1.30)	<0.001
Charlson Comorbidity Index	0.71 (0.74)	1.26 (0.68)	<0.001
Grip strength (kg)	17.65 (6.59)	12.90 (4.85)	<0.001
Barthel Index	57.28 (24.14)	22.93 (24.93)	<0.001
Gait speed (m/s)	0.68 (0.33)	0.49 (0.28)	<0.001
Unable to walk, No. (%)	46 (26.59)	96 (72.18)	<0.001
MMSE	23.33 (6.07)	14.60 (7.77)	<0.001
SMI (kg/m^2^)	5.79 (1.15)	4.68 (1.35)	<0.001
BMI (kg/m^2^)	21.01 (3.38)	16.90 (2.79)	<0.001
Lingual pressure (kPa)	27.99 (6.33)	21.53 (6.15)	<0.001
Malnutrition, No. (%)	81 (46.29)	125 (93.98)	<0.001
History of stroke, No. (%)	23 (13.29)	34 (25.56)	0.012
FOIS	6.60 (0.49)	3.84 (1.60)	<0.001
Primary disease			<0.001
Orthopedics, No. (%)	130 (74.29)	47 (35.34)	
Heart failure, No. (%)	12 (6.86)	9 (6.77)	
Digestive disorder, No. (%)	8 (4.57)	10 (7.52)	
Urologic disease, No. (%)	4 (2.29)	7 (5.26)	
Pneumonia, No. (%)	6 (3.43)	34 (25.56)	
Others, No. (%)	15 (8.57)	26 (19.55)	

Variables are presented as mean (SD), unless indicated as No. (%). Abbreviations: BMI, body mass index; FOIS, Functional Oral Intake Scale; MMSE, Mini-Mental State Examination; SMI, Skeletal Muscle Mass Index.

**Table 2:** Estimated odds ratios of the tested models for sarcopenic dysphagia.

	Model 1	Model 2	Model 3	Image-Only Model
Intercept	0.42	0.48	0.47	0.89
	(0.24–0.76)	(0.27–0.88)	(0.26–0.85)	(0.67–1.19)
Median pixel value	1.08	1.08	—	1.19
	(0.75–1.55)	(0.74–1.56)		(0.86–1.64)
IQR of pixel values	1.30	1.27	1.28	1.29
	(0.95–1.78)	(0.92–1.76)	(0.93–1.77)	(0.96–1.73)
Number of feature points	1.65	1.55	1.61	1.86
	(1.06–2.56)	(0.99–2.41)	(1.07–2.41)	(1.22–2.86)
Age	—	1.59	1.59	—
		(1.14–2.23)	(1.14–2.23)	
Sex: female	0.69	0.55	0.57	—
	(0.36–1.35)	(0.27–1.12)	(0.28–1.13)	
BMI: not underweight	reference	reference	reference	—
BMI: underweight	6.43	6.24	6.33	—
	(3.37–12.24)	(3.24–12.05)	(3.29–12.18)	

Variables are presented as odds ratios (95% confidence interval). Model 1: sex, categorized BMI, median pixel value, IQR of pixel value, number of feature points. Model 2: age, sex, categorized BMI, median pixel value, IQR of pixel value, number of feature points. Model 3: age, sex, categorized BMI, IQR of pixel value, number of feature points. Image-only model: median pixel value, IQR of pixel value, number of feature points. Abbreviations: BMI, body mass index; IQR, interquartile range.

**Table 3:** Prediction performances of the screening models.

	ROC-AUC	Se (%)	Sp (%)	PPV (%)	NPV (%)	PR-AUC
Model 1	0.876	75.00	85.00	72.73	86.44	0.838
Model 1 w/o image features	0.811	75.00	83.33	70.59	86.21	0.683
Model 2	0.877	87.50	76.67	66.67	92.00	0.838
Model 2 w/o image features	0.832	87.50	73.33	63.64	91.67	0.670
Model 3	0.871	87.50	73.33	63.64	91.67	0.816
Image-only model	0.814	71.88	80.00	65.71	84.21	0.726

Model 1: sex, categorized BMI, median pixel value, IQR of pixel value, number of feature points. Model 2: age, sex, categorized BMI, median pixel value, IQR of pixel value, number of feature points. Model 3: age, sex, categorized BMI, IQR of pixel value, number of feature points. Image-only model: median pixel value, IQR of pixel value, number of feature points. Abbreviations: NPV, negative predictive value; PPV, positive predictive value; PR-AUC, area under the curve of precision-recall curve; ROC-AUC, area under the curve of the receiver operating characteristic curve; Se, sensitivity; Sp, specificity; w/o, without.

## Data Availability

Data sharing is not applicable to this article.
